# Risk mitigation of shared room ventilation and filtration on SARS-CoV-2 transmission: a multicenter test-negative study

**DOI:** 10.1017/ice.2025.10256

**Published:** 2025-10

**Authors:** Victoria Williams, Kevin L. Schwartz, Kevin Brown, Matthew Muller, Jeff Powis, Daniel Ricciuto, Alexander Kiss, Mark Downing, Sharon Sukhdeo, Thomas Dashwood, Jacob Romano, Rob Kozak, Lorraine Maze dit Mieusement, Jerome A. Leis

**Affiliations:** 1 Sunnybrook Health Sciences Centre, Toronto, ON, Canada; 2 St. Joseph’s Health Centre, Unity Health Toronto, Toronto, ON, Canada; 3 Public Health Ontario, ON, Canada; 4 Dalla Lana School of Public Health, University of Toronto, Toronto, ON, Canada; 5 Temerty Faculty of Medicine, University of Toronto, Toronto, ON, Canada; 6 St. Michael’s Hospital, Unity Health Toronto, Toronto, ON, Canada; 7 Michael Garron Hospital, Toronto, ON, Canada; 8 Lakeridge Health, Oshawa, ON, Canada; 9 Centre for Quality Improvement and Patient Safety, University of Toronto, Toronto, ON, Canada

## Abstract

**Background:**

Admission to shared hospital rooms are a risk factor of healthcare-associated (HA) SARS-CoV-2. Quantifying the impact of engineering controls such as ventilation and filtration is essential to informing resource utilization and infection prevention guidelines.

**Methods:**

Multicenter test-negative study of patients exposed to SARS-CoV-2 in shared rooms across five hospitals between January and October, 2022. Independent variables tested were measured air changes per hour (ACH), presence of any room mechanical ventilation (RMV), or portable high-efficiency particulate air (HEPA) filter. Covariates included facility (number of beds in room, outbreak status of unit), source patient (presence of symptoms, RT-PCR cycle threshold (Ct) value), and exposed patient factors (age, sex, time from last SARS-CoV-2 vaccine, previous SARS-CoV-2 infection, exposure duration). Multilevel logistic mixed models used to estimate the impact of engineering controls on transmission.

**Results:**

Among 468 exposed patients, secondary attack rate was 26.3% (range 7.5–33.3% across hospitals). In multivariable analysis, increased ACH was associated with decreased odds of infection (adjusted odds ratio (aOR) 0.88, 95% CI 0.78–1.00; p=.046) as were exposure duration and Ct value of source patient. Presence of RMV was also associated with decreased odds of infection (aOR 0.51, 95% CI 0.27–0.95; p=.034) while use of portable HEPA filter was not significant (aOR 0.58, 95% CI 0.26–1.31; p=.18).

**Conclusions:**

Improved ventilation was independently associated with lower odds of SARS-CoV-2 infection among exposed roommates. Ensuring RMV is present and optimizing ACH may significantly mitigate the risk of HA-SARS-CoV-2. Future prospective studies should assess optimal ACH thresholds and the impact of portable HEPA filters.

## Background

Transmission of Severe Acute Respiratory Syndrome Coronavirus 2 (SARS-CoV-2), either through inhalation, deposition, or contact, contributes significantly to the burden of healthcare-associated (HA) viral respiratory infection (VRI).^1,2^

Admission to shared hospital rooms is a known risk factor of HA-SARS-CoV-2 arising from pre-symptomatic and asymptomatic roommates.^2^ Retrospective studies throughout the COVID-19 pandemic revealed secondary attack rates in shared rooms that ranged from 22 to 39%, similar to that seen in household contacts (range 17 to 32%).^3–10^ Longer exposure duration, lower real-time polymerase chain reaction (RT-PCR) cycle threshold (Ct) value (suggesting higher viral load), and increased number of patients per room significantly increased transmission risk in prior studies.^3,4,9,11^

Ventilation and filtration of infectious respiratory particles is believed to mitigate the risk of transmission of respiratory viruses but has not been frequently measured in prior roommate studies.^4,5,9,10,12^ Many hospitals have invested in optimizing air changes per hour (ACH) of existing heating, ventilation, and air-conditioning (HVAC) systems and/or placing portable high-efficiency particulate air (HEPA) filters between beds.

Quantifying the impact of such engineering controls on transmission of respiratory viruses is essential to informing resource utilization and infection prevention guidelines. In this multicenter study, our objective was to determine the association between room ventilation and filtration on the risk of SARS-CoV-2 transmission in shared hospital rooms during the COVID-19 pandemic as Omicron variants were circulating.

## Methods

### Study design

We performed a multicenter test-negative study of patients exposed to SARS-CoV-2 in shared rooms across five acute care hospitals in Ontario, Canada, between January 1, 2022, and October 31, 2022 (when the Omicron variant was dominant). As a test-negative design, the cohort of exposed patients was recruited before their case status was known, then analyzed as a case-control study nested within this cohort.^13^ All ward-level acute care inpatient units with four-walled rooms at the participating hospitals were included while open pod care units were excluded. All participating hospitals received research ethics board approval from their local institutional review board. The need for informed consent to be included was waived.

### Setting

During the study period, universal admission screening for SARS-CoV-2 was in place at all participating hospitals, and some periodic point prevalence testing. All sites had universal masking policies for healthcare workers (N95 respirator or medical mask) in patient care areas. Optimization of ACH and/or addition of HEPA filters occurred in some inpatient units prior to and early in the COVID-19 pandemic, but no additional changes were made during the study period.

All patients routinely underwent surveillance for development of VRI symptoms. Midturbinate or nasopharyngeal swabs were obtained by trained nursing staff and processed using an RT-PCR test that detects SARS-CoV-2 E, UTR, and N genes.^14,15^ Source and exposed patients were placed in transmission-based precautions (private room, with gown, gloves, N95 respirator, and eye protection used by healthcare workers) for the duration of the period of communicability and follow-up period, respectively.

Exposed patients were tested upon detection of SARS-CoV-2 in the source patient and up to day 10 post-exposure on a schedule that differed by hospital but included a minimum of one additional asymptomatic test prior to day 10. Additional testing was completed if the patient developed symptoms of VRI during this timeframe.

### Participants

Patients eligible for inclusion were roommates (exposed) to patients with laboratory-confirmed SARS-CoV-2 (source), defined as those who shared a room for a minimum of 15 minutes during the source patient’s period of communicability. The period of communicability was defined as 24 hours prior to and up to 10 days after symptom onset. In the absence of symptoms in the source patient, the period of communicability was determined to begin on the date that the specimen was obtained for SARS-CoV-2 testing.

Exposed patients were excluded for any of the following reasons: a confirmed history of SARS-CoV-2 infection within 90 days preceding the exposure; an additional exposure to a healthcare worker or visitor with a confirmed SARS-CoV-2; lacking a negative SARS-CoV-2 test at baseline (ie after the source patient’s symptom onset or test positivity); testing positive for SARS-CoV-2 within 72 hours of the onset of symptoms and/or test positivity in the source patient; or receiving incomplete post-exposure follow-up testing (defined as a minimum of 5 days of observation).

### Variables

The primary outcome was the odds of infection with SARS-CoV-2 following roommate exposure. A case was defined as an exposed patient who tested negative for SARS-CoV-2 after identification of the source patient and subsequently tested positive 72 hours or more after the onset of symptoms/test date in the source patient (based on a mean incubation of 3.42 days for the Omicron variant) and up to 10 days following the end of the exposure.^16^ Exposed patients were assigned as controls if they consistently tested negative for SARS-CoV-2, including at least one negative test between day 5 and 10 after the end of the exposure. Secondary attack rate (SAR) overall and by hospital was also calculated, defined as the incidence of laboratory-confirmed SARS-CoV-2 among exposed patients.

The independent (exposure) variable was the ventilation and/or filtration in the room where exposure occurred. This variable was assessed in three different ways. First, total measured ACH was assessed, defined as the number of times per hour the entire volume of air in a given space is replaced with supply and/or recirculated air. Exposures where the ACH was estimated or unmeasured were excluded. Second, presence of room mechanical ventilation (RMV) was assessed, regardless of whether or not ACH measurements were available. RMV was considered present if the room was supplied by a central building HVAC system with air supply/exhaust vents or room-level modifications that created RMV (eg installation of HEPA unit exhausted externally at Hospital 2). Third, presence of a portable HEPA for filtration was assessed, defined as the documented presence or absence of a portable HEPA filtration unit exhausted within the room at the time the exposure occurred. One hospital (Hospital 5) did not have stable designated room locations for its portable HEPA filtration units and therefore was excluded from this analysis.

Additional pre-specified covariates included facility level (number of beds in the room, outbreak status of the unit at time of exposure), source patient level (presence of symptoms, Ct value), and exposed patient level (age, sex, time from last SARS-CoV-2 vaccine, history of SARS-CoV-2 infection >90 days preceding the exposure, duration of exposure in hours). An outbreak was defined as 2 or more patients who are epidemiologically linked, both with positive results within a 7-day period.^17^ An outbreak would not be triggered when the second case is a current or former roommate of a known case and there is no evidence of uncontrolled transmission in the area.

### Data sources

All patients exposed to SARS-CoV-2 were identified and followed by Infection Prevention and Control (IPAC) practitioners as part of routine hospital surveillance. Trained data abstractors obtained additional source and exposure patient data from laboratory information systems, hospital IPAC databases, and patient healthcare records. Exposure variables were obtained from plant operations and IPAC records available preceding the start of the study period.

### Study size

During the planning phase of this retrospective study, we assessed that to identify a 20% difference in the odds of transmission, while accounting for 10 predictors in the regression model, a sample size of 500 participants would be needed to achieve a power of 80% and alpha of 5%. This number corresponded to the minimum number of shared-room exposures across the five hospitals during the course of the study period, before applying the pre-specified exclusion criteria.

### Statistical methods

Continuous variables were measured with mean and standard deviation while categorical variables were assessed by proportions with 95% confidence intervals. To evaluate the impact of the exposure variable, ventilation/filtration, a logistic mixed model was run with case (1) or control (0) status as the binary outcome and source patients and hospitals as two separate levels of random intercepts. Unadjusted models included no covariates, while adjusted models included specified covariates as fixed effects. Three models were built with first measured ACH (model 1), then presence of any RMV (model 2), and then the presence of HEPA filters used for filtration (model 3) as the primary exposure variable. To estimate the odds of SARS-CoV-2 infection based on the continuous variable of ACH, the means of other covariates in the model were used to generate exponentiated estimates of the odds of secondary transmission.

A sensitivity analysis was performed where the case definition was changed from 72 hours after to 24 hours or more after the onset of symptoms (or test date if asymptomatic) of the source patient. The data is presented as odds ratios (OR) with 95% confidence intervals (CI), and *p*<0.05 considered statistically significant. Analyses were carried out using SAS Version 9.4 (SAS Institute, Cary, NC, USA).

## Results

There were a total of 1007 patient exposures associated with 484 source patients identified during the study period. Among these patient exposures, 468 (46.4%) were eligible for inclusion, associated with 406 source patients.

**Figure 1.**
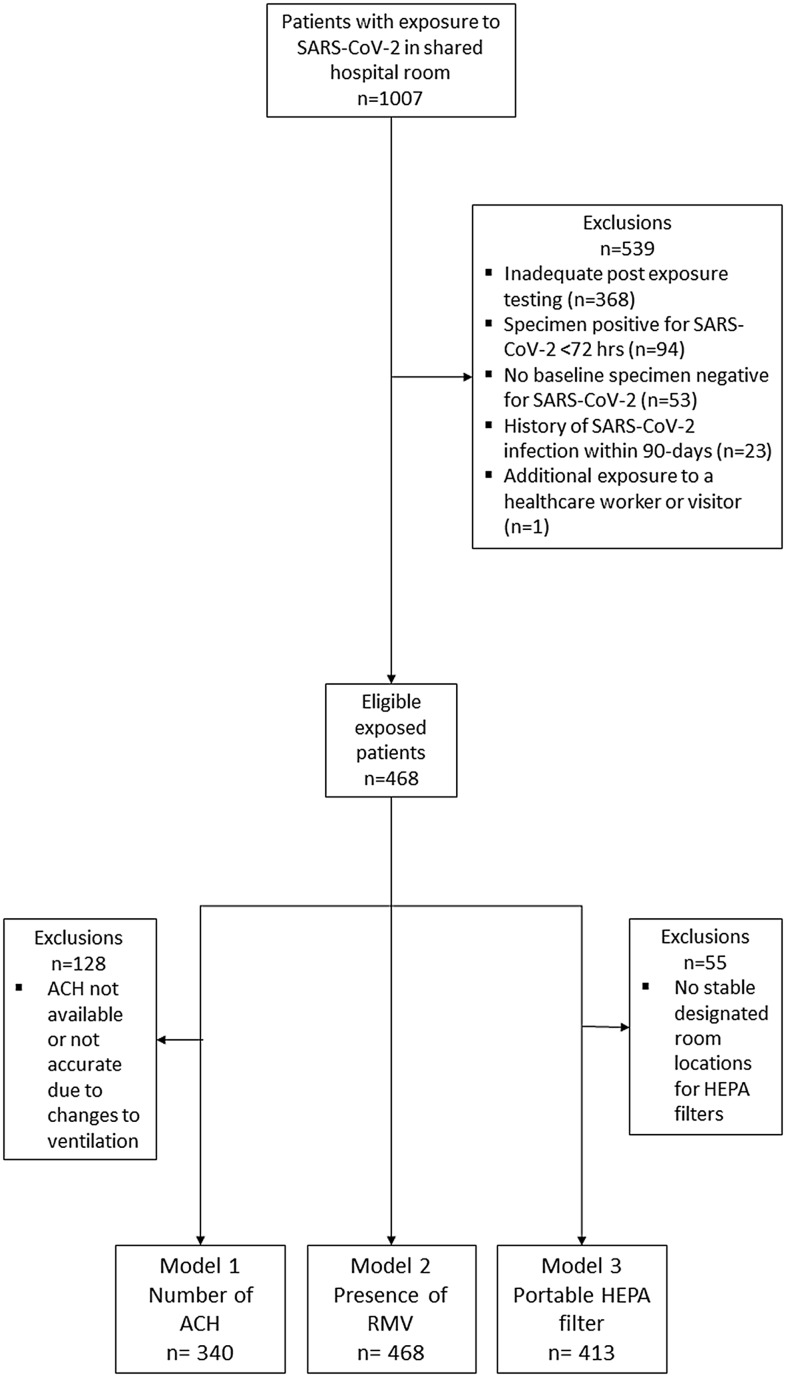
Study flow diagram of eligible patients exposed to SARS-CoV-2 in shared hospital rooms. ACH=Air changes per hour, RMV=Room mechanical ventilation, HEPA=high-efficiency particulate air.

Figure 1 depicts the study flow diagram. There were 539 exposed patients excluded due to inadequate post-exposure follow-up testing (n=368), testing positive for SARS-CoV-2 within 72 hours of the source patient (n=94), no specimen negative for SARS-CoV-2 post-exposure prior to positive test (n=53), history of SARS-CoV-2 infection within 90 days of the exposure (n=23), or additional exposure to a healthcare worker or visitor with confirmed SARS-CoV-2 infection (n=1).

Table 1 describes the baseline characteristics of the exposed patient cohort across the five hospitals. Among 468 exposed patients, there were 123 cases and 345 controls. The SAR was 26.3% (123/468) and ranged from 7.5 to 33.3% across the five hospitals.

**Table 1.** Baseline characteristics of patients admitted to shared rooms and exposed to a roommate with SARS-CoV-2, across five acute care hospitals between January and October 2022

	All sites	Hospital 1	Hospital 2	Hospital 3	Hospital 4	Hospital 5
**Number of exposed patients**	468	132	121	120	40	55
**Number of cases (SAR)**	123(26.3)	30(22.7)	33(27.3)	40(33.3)	3(7.5)	17(30.9)
**Exposed patient age (mean yrs** ±**SD)**	75.8±14.3	74.5±14.2	77.5±13.8	77.2±12.5	69.0±17.6	76.7±15.5
**Exposed patient sex (% male)**	239(51.1)	70(53.0)	59(48.8)	53(44.2)	26(65.0)	31(56.4)
**Exposed patient months since last vaccine dose (mean** ±**SD)**	5.0±3.6	5.6±3.5	4.9±4.1	3.7±2.9	5.4±3.6	6.4±2.8
**Exposed patient had history of SARS-CoV-2^^^(%)**	43(9.2)	12(9.1)	10(8.3)	10(8.3)	2(5.0)	9(16.4)
**Exposure duration (mean hrs** ±**SD)**	27.1±22.3	21.9±13.9	20.3±15.0	37.7±30.8	30.0±27.7	29.2±16.5
**Source patient symptomatic (%)**	214(45.9)	80(60.6)	43(36.1)	47(39.2)	21(52.5)	23(41.8)
**Source patient Ct value (mean** ±**SD)**	23.9±6.7	22.2±6.9	25.2±5.5	24.2±7.1	26.6±7.0	23.3±7.2
**Outbreak on unit (%)**	157(33.5)	36(27.3)	20(16.5)	55(45.8)	7(17.5)	16(29.1)
**Number of beds in the room (mean** ±**SD)**	2.8±0.9	2.7±0.5	2.6±0.6	3.4±1.2	3.1±1.0	2.5±0.8
**Air changes per hour (mean** ±**SD)**	3.2±2.7	4.2±2.4	–	3.9±2.3	6.0±0.0	0.95±2.3
**Room mechanical ventilation (%)**	369(78.8)	132(100)	82(67.8)	107(89.2)	40(100)	8(14.5)
**Portable HEPA for filtration (%)**	66(16.0)	34(25.8)	0(0)	32(26.7)	0(0)	–

^ Patients with SARS-CoV-2 infection within 90 days were excluded. SAR=secondary attack rate, SD=Standard Deviation, Ct=Cycle threshold, HEPA=high-efficiency particulate air.

**Figure 2.**
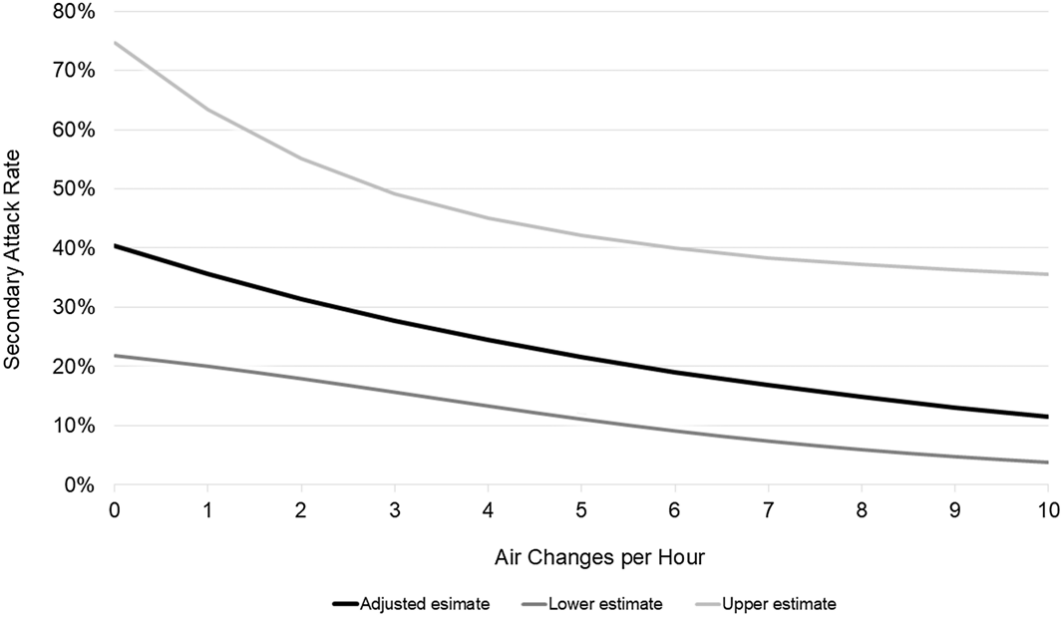
Estimated SARS-CoV-2 secondary attack rate along with 95% confidence intervals in shared hospital room, per number of room air changes per hour (ACH) after adjustment for other confounders.

Increased ACH was associated with decreased odds of infection in the unadjusted model (OR 0.89, 95% CI 0.80–0.98; p=.018). In the multivariable analysis, ACH remained significant (adjusted odds ratio, aOR 0.88, 95% CI 0.78–1.00; p=.046) as were exposure duration and the Ct value of the source patient (Table 2). The estimated SAR, after adjusting for covariates, ranged from 40% for ACH less than 1, to <20% for ACH >6 (Figure 2).

**Table 2.** Association between measured air changes per hour (ACH), presence of room mechanical ventilation (RMV), and presence of portable HEPA (high-efficiency particulate air) filters in shared hospital rooms, on transmission of SARS-CoV-2 to exposed roommates

Risk Factor	Model 1 (ACH) n=340		Model 2 (RMV) n=468		Model 3 (HEPA) n=413	
	OR (95% CI)	p-value	OR (95% CI)	p-value	OR (95% CI)	p-value
**Air changes per hour**	**0.88** **(0.78**–**1.00)**	**.046**	–	–	–	–
**Room mechanical ventilation**	–	–	**0.51** **(0.27**–**0.95)**	**.034**	–	–
**Portable HEPA for filtration**	–	–	–	–	0.58 (0.26–1.31)	.18
**Exposed patient age (yrs)**	0.99 (0.96–1.01)	.30	1.00 (0.98–1.02)	.84	1.00 (0.98–1.02)	.96
**Exposed patient sex (male)**	0.60 (0.31–1.15)	.12	0.74 (0.44–1.25)	.25	0.64 (0.36–1.12)	.11
**Exposed patient months since last vaccine dose**	0.96 (0.87–1.06)	.41	0.99 (0.92–1.06)	.67	0.96 (0.89–1.04)	.35
**Exposed patient had history of SARS-CoV-2 infection^^^**	0.52 (0.17–1.64)	.26	0.65 (0.25–1.65)	.35	0.97 (0.36–2.61)	.95
**Exposure duration (hrs)**	**1.01** **(1.00–1.03)**	**.036**	**1.01** **(1.00–1.03)**	**.030**	**1.01** **(1.00–1.03)**	**.039**
**Number of beds in the room**	1.29 (0.94–1.77)	.11	**1.34** **(1.01–1.77)**	**.043**	**1.40** **(1.03–1.89)**	**.033**
**Outbreak on unit**	1.39 (0.73–2.66)	.30	1.25 (0.72–2.17)	.42	1.15 (0.62–2.15)	.65
**Source patient Ct value**	**0.95** **(0.90–0.99)**	**.031**	0.96 (0.92–1.00)	.064	0.96 (0.92–1.00)	.058
**Source patient symptomatic**	0.80 (0.41–1.58)	.51	0.99 (0.57–1.73)	.98	0.98 (0.54–1.78)	.95

^ Patients with SARS-CoV-2 infection within 90 days were excluded. OR=odds ratio, CI=confidence interval, ACH=air changes per hour, RMV=room mechanical ventilation, HEPA=high-efficiency particulate air.

The presence of RMV was associated with decreased odds of infection in the unadjusted analysis (OR 0.49, 95% CI 0.29–0.83; p=.009) and remained significant in the adjusted model (aOR 0.51, 0.27–0.95; p=.034). In this model, increased exposure duration and number of beds in the shared room were also associated with increased odds of infection (Table 2).

The use of HEPA for filtration was not associated with lower odds of infection in unadjusted (OR 0.92, 95% CI 0.47–1.81; p=.81) or adjusted analysis (aOR 0.58, 95% CI 0.26–1.31; p=.18). Once again, longer duration of exposure and more beds in the room were associated with a higher odds of infection (Table 2).

In the sensitivity analysis shortening the case definition to 24 hours after exposure, there were 511 (50.7%) eligible exposed patients with secondary attack rate of 32.5% (166/511) that ranged from 11.9 to 42.0% across the five hospitals. The results were similar with increased ACH remaining significantly associated with reduced odds of infection in shared rooms (aOR 0.89, 95% CI 0.80–1.00; p=.042), while RMV (aOR 0.58, 95% CI 0.33–1.02; p=.059) and use of HEPA filters (aOR 0.61, 95% CI 0.31–1.23; p=.16) were not significant (Table 3).

**Table 3.** Sensitivity analysis where case definition of exposed patients lowered to 24 hours or more after onset of symptoms/test date of the source patient

Risk Factor	Model 1 (ACH) n=369		Model 2 (RMV) n=511		Model 3 (HEPA) n=451	
	OR (95% CI)	p-value	OR (95% CI)	p-value	OR (95% CI)	p-value
**Air changes per hour**	**0.89 (0.80–1.00)**	**.042**	–	–	–	–
**Room mechanical ventilation**	–	–	0.58 (0.33–1.02)	.059	–	–
**Portable HEPA for filtration**	–	–	–	–	0.61 (0.31–1.23)	.16
**Exposed patient age (yrs)**	0.98 (0.96–1.01)	.14	0.99 (0.98–1.01)	.37	0.99 (0.97–1.01)	.41
**Exposed patient sex (male)**	**0.53 (0.29–0.96)**	**.038**	0.68 (0.43–1.08)	.10	0.63 (0.38–1.03)	.064
**Exposed patient months since last vaccine dose**	0.97 (0.89–1.06)	.50	1.01 (0.94–1.07)	.89	0.99 (0.93–1.06)	.86
**Exposed patient had history of SARS-CoV-2 infection^^^**	0.39 (0.13–1.18)	.093	0.44 (0.18–1.10)	.078	0.61 (0.23–1.60)	.31
**Exposure duration (hrs)**	**1.01 (1.00–1.03)**	**.031**	**1.01 (1.00–1.03)**	**.014**	**1.01 (1.00–1.03)**	**.017**
**Number of beds in the room**	1.25 (0.94–1.67)	.12	1.26 (0.98–1.61)	.072	1.30 (0.99–1.70)	.055
**Outbreak on unit**	**1.89 (1.05–3.38)**	**.034**	**1.65 (1.03–2.67)**	**.040**	1.57 (0.93–2.67)	.091
**Source patient Ct value**	**0.94 (0.89–0.98)**	**.0081**	**0.96 (0.93–1.00)**	**.031**	**0.96 (0.92–1.00)**	**.035**
**Source patient symptomatic**	0.65 (0.35–1.22)	.17	0.89 (0.54–1.47)	.65	0.90 (0.53–1.52)	.68

^ Patients with SARS-CoV-2 infection within 90 days were excluded. OR=odds ratio, CI=confidence interval, ACH=air changes per hour, RMV=room mechanical ventilation, HEPA=high-efficiency particulate air.

## Discussion

In this multicenter test-negative case-control study, improved ventilation was associated with reduced odds of infection of SARS-CoV-2 among patients exposed in a shared hospital room. For each additional ACH, we measured an estimated 12% lower odds of infection, while presence of any RMV carried approximately 50% lower odds. The effect size of the presence of a portable HEPA filtration units placed in shared rooms was similar; however, confidence intervals were wide, and this finding was not statistically significant.

The protective role of ventilation has been widely recognized for over two decades with international guidelines recommending optimization of engineering controls in healthcare environments.^18,19^ Despite this, few systematically designed studies assessed the quantitative effect of ventilation and/or filtration on transmission of respiratory viruses prior to 2019.^20^ During the COVID-19 pandemic, multiple outbreak investigations and aerodynamic studies suggested that a lack of ventilation can facilitate transmission of SARS-CoV-2, and improved ventilation with or without filtration can improve clearance of environmental air samples.^21–25^

There are comparatively few studies that systematically quantified the impact of these engineering controls on SARS-CoV-2 transmission risk especially after adjustment for confounding factors. A single-site case-control study of hospitalized roommates exposed to SARS-CoV-2 found no association between median ACH (OR 0.76, 95% CI 0.46–1.27), but was limited to a small sample size of 37 exposed roommates.^3^ More recently, a prospective study of resident contacts in congregate care facilities similarly found 10% lower risk in SARS-CoV-2 transmission associated with increased ACH in an adjusted analysis.^26^

Our multicenter study undertook a systematic approach to quantifying the relative impact of ventilation and filtration in modulating the risk of transmission among hospitalized roommates. The unadjusted, adjusted, and sensitivity analyses all consistently revealed an estimated 12% lower odds of SARS-CoV-2 transmission for every additional ACH, which could significantly improve the safety of shared room environments, above and beyond other existing mitigation measures.

Current standards in both the United States and Canada that define ventilation system design requirements in health care facilities recommend that hospital ward-level rooms have a minimum of 6 total ACH.^27,28^ The variability across the five participating hospitals reflects hospital designs that pre-dated these standards. Despite some of the improvements already made to older infrastructure, our findings support additional investment in optimizing ventilation in shared rooms to improve patient safety given the recognized risk of healthcare-associated viral respiratory infections.^3–10^

In situations where ACH cannot be further optimized, HEPA filtration has historically been used to attempt to mitigate this risk, yet clinical evidence assessing the impact of portable HEPA filters is lacking. Multiple experimental studies suggest HEPA filtration units can accelerate clearance of infectious respiratory particles.^21,22,29^^–^^31^ In one simulation study, operation of a portable HEPA filter reduced transfer to the bed adjacent to the outlet vent but did not offer a benefit over closing the curtains alone.^21^ In our study, the lack of a statistically significant association between HEPA filters and transmission of SARS-CoV-2 may be due to several factors. First, the sample size was smaller than that for the other two exposure variables as HEPA filters were only present during a proportion of exposures in two facilities accounting for only 16% of all patient exposures. Second, our study could not reliably assess whether HEPA filters remained in operation or in appropriate placement throughout patient exposures.

The optimal threshold for ACH to minimize risk of transmission of SARS-CoV-2 in shared hospital rooms or other respiratory viruses remains undefined. Our estimated secondary attack rate per ACH after adjustment for other covariates suggests the potential for additional risk reduction with ventilation exceeding current standards. A key lesson of the COVID-19 pandemic was the need for higher-quality evidence to evaluate non-pharmacological interventions (NPI).^32–34^ Future research on the impact of ventilation and filtration in healthcare settings on patient outcomes should include pragmatic prospective study designs that can better characterize the optimal engineering thresholds.

Our study has several strengths. First, it was conducted across multiple hospitals with standardized application of case and control definitions and strict eligibility criteria requiring complete follow-up. While study criteria were applied universally across the five hospitals, differences in the infrastructure of the facilities increases generalizability of the findings. Second, we adjusted for multiple potential confounders including factors previously identified to be significant in prior hospital roommate studies such as duration of exposure, number of beds, and Ct value.^3,4,9^ Third, the use of multilevel logistic mixed models adjusted for clustering within source patients and hospitals. Finally, we explored different exposure variables to assess both ventilation and filtration in different ways.

Our study also has important limitations. First, as test-negative study we cannot exclude the possibility of unmeasured confounders. We defined the source as the patient but cannot rule out unrecognized exposures to SARS-CoV-2, such as visitors and healthcare workers. We did not adjust for room size or distance between beds, which could also be important confounders. Second, although the patient exposure was determined epidemiologically and all exposed patients required a negative baseline test to be included, whole genome sequencing analysis was not used to confirm these transmission events. Third, our study assessed the impact of total ACH and due to the possibility of recirculated air, the effect of fresh ACH may be different.

Across five hospitals, improved ventilation in shared rooms was independently associated with lower odds of SARS-CoV-2 infection among exposed roommates. Ensuring RMV is present and optimizing ACH may significantly mitigate the risk of HA-SARS-CoV-2. Future prospective NPI trials should assess the optimal ACH thresholds and the impact of portable HEPA filters.

## Data sharing statement

The individual patient data are not permitted to be shared under the current approved protocol but the data dictionary that defines each field in the data set and the analytic code used for the analysis will be made available to other research groups upon request. Such requests should provide evidence to support the request for access along with ethics approval for the request.
